# Cross-reacting antiporcine factor VIII inhibitors in patients with acquired hemophilia A

**DOI:** 10.1016/j.rpth.2024.102553

**Published:** 2024-08-22

**Authors:** Maddie Stephen, Carolyne Elbaz, Hina Hanif, Katerina Pavenski, Jerry Teitel, Michelle Sholzberg

**Affiliations:** 1 Department of Medicine, Division of Hematology, University of Toronto, Toronto, Ontario, Canada; 2 Department of Medicine, Division of Hematology/Oncology, St. Michael’s Hospital, University of Toronto, Toronto, Ontario, Canada; 3 Department of Laboratory Medicine and Pathobiology, St. Michael’s Hospital, University of Toronto, Toronto, Ontario, Canada

**Keywords:** acquired hemophilia A, inhibitors, Obizur, rpFVIII

## Abstract

• Recombinant porcine factor VIII (rpFVIII) is a hemostatic treatment for bleeding in acquired hemophilia A.
• The efficacy of rpFVIII can be negatively affected by cross-reacting anti-human FVIII antibodies.
• In this cohort, 52% of patients with acquired hemophilia A had baseline cross-reacting antibodies.
• Cross-reacting antibodies were associated with decreased FVIII activity levels post-rpFVIII infusion.

## 1 Introduction

Acquired hemophilia A (AHA) is a rare autoimmune blood disorder characterized by the development of autoantibodies against endogenous factor (F)VIII. These antibodies, also called inhibitors, result in spontaneous or trauma-induced bleeding in patients without a history of bleeding. Risk factors for AHA include advanced age, malignancy, autoimmune diseases, and pregnancy or the postpartum period, although 50% of cases are idiopathic [1].

Patients with AHA often require hemostatic therapy with bypassing agents such as recombinant FVIIa and activated prothrombin complex concentrate or porcine FVIII concentrate. Plasma-derived porcine FVIII concentrate (Hyate:C [Ipsen]) was removed from the market in 2004 due to viral safety concerns [2,3]. Recombinant B-domain-deleted porcine FVIII (rpFVIII) is licensed for treatment of acute bleeding in patients with AHA in Canada, Europe, the United States, and Australia. Structural differences in the A2 and C2 domains reduce the reactivity of rpFVIII with antihuman FVIII (anti-hFVIII) inhibitors, allowing it to provide cofactor activity for FIX in the human intrinsic tenase reaction [4]. However, the hemostatic efficacy of rpFVIII can be disrupted if anti-hFVIII antibodies cross-react with rpFVIII.

A prospective phase 2/3 multicenter trial described the efficacy and safety of rpFVIII for the treatment of serious bleeds in patients with AHA. In this study, cross-reacting anti-rpFVIII antibodies were found in 35% of subjects prior to initiation of rpFVIII, and *de novo* allo-anti-rpFVIII antibodies were described in 18% of patients who received rpFVIII [5].

Our prior experience with anti-rpFVIII in patients with congenital and acquired HA was presented in 2019 at the American Society of Hematology conference [6]. A total of 61% of patients had detectable anti-rpFVIII inhibitors prior to rpFVIII exposure, and 13% of patients with no detectable baseline anti-rpFVIII antibodies developed a *de novo* inhibitor after rpFVIII exposure [6]. The primary objective of the current study was to extend the previously published dataset [6] to include 30 additional patients with AHA. We aimed to describe the frequency of baseline cross-reacting rpFVIII inhibitors in patients with AHA. Our secondary goal was to analyze the effect of baseline rpFVIII antibodies on FVIII recovery after treatment with rpFVIII and to describe the changes in anti-rpFVIII titers and *de novo* inhibitor formation after exposure to rpFVIII.

## 2 Methods

This was a retrospective single-center study carried out between 2016 and 2021 at St. Michael's Hospital, home to the largest Canadian Hemophilia Treatment Centre in Toronto, Canada. Institutional research ethics board approval was obtained. Electronic charts of patients admitted to our institution with AHA who underwent testing for rpFVIII inhibitors were reviewed. Demographic, relevant clinical, and laboratory variables were collected in a deidentified fashion. Descriptive statistics (median, range, and IQR) were used.

FVIII activity assay, anti-hFVIII Bethesda assay, and the rpFVIII inhibitor assay were performed in the special coagulation laboratory at St. Michael’s Hospital. The rpFVIII inhibitor assay was performed using the Nijmegen-modified Bethesda assay [7]. The patient sample is initially heat-treated at 57 °C for 30 minutes to remove residual exogenous FVIII. The substrate is a mixture of freeze-dried concentrate of rpFVIII porcine sequence in a mixture of stabilizing excipients. This assay increases the specificity of low-titer FVIII inhibitor measurements by buffering the normal pooled plasma, used in patient and control mixtures, to pH 7.4 with imidazole buffer and using congenital FVIII-deficient plasma in the control mixture and for preparing patient dilutions. Both adjustments serve to maintain the pH of the reaction mixtures for the 2-hour incubation period and thereby stabilize FVIII in the normal pooled plasma [8].

Baseline cross-reacting inhibitors were defined as those present prior to rpFVIII exposure.

## 3 Results

### 3.1 Clinical findings

Fifty patients underwent testing for porcine inhibitors since assay availability in 2016 at St. Michael’s Hospital. Most patients (96%) presented with bleeding, and the most common sites were mucocutaneous and deep tissue bleeding (eg, intramuscular hematoma). Nineteen patients presented with more than 1 type of bleeding (Table 1).

**Table 1:** Baseline demographic, clinical, and laboratory characteristics.

Demographic data	Number of patients (Total *N* = 50)
**Characteristic**	**All patients (*N* = 50)**
Female, *n* (%)	15 (30)
**Clinical data**	
**Types of bleeds, *n* (%)**	
Mucocutaneous and deep tissue	40 (80)
Gastrointestinal	7 (14)
Hematuria	2 (4)
Hemarthrosis	0 (0)
Intracranial	2 (4)
Postoperative	1 (2)
None	2 (4)
More than 1 type of bleed	19 (38)
**Laboratory data**	
Baseline FVIII activity in IU/mL, median (IQR)	0.02 (0.01-0.08)
Anti-hFVIII in BU/mL, median (IQR)	54 (14-147)
**Patients with detectable anti-rpFVIII**	
AHA, *n* (%)	26 (52)
Anti-rpFVIII inhibitor in pBU/mL, median (IQR)	2 (0.86-5.8)
**FVIII activity level response in patients who received rpFVIII**	
**Variable**	**Total *N* = 50**
No. of patients who received rpFVIII, *n* (%)	42 (84)
No. of patients with anti-rpFVIII who received rpFVIII	22
Average (range) first treatment dose (U/kg)	99 (5.9-155)
Median (range) recovery in FVIII activity after first dose (IU/mL per U/kg infused)	1.2 (0-2.5)

AHA, acquired hemophilia A; BU, Bethesda Units; FVIII, factor VIII; hFVIII, human factor VIII; rpFVIII, recombinant B-domain-deleted porcine factor VIII.

### 3.2 Laboratory results

Among all 50 patients tested, the median baseline FVIII activity was 0.02 IU/mL, with a median anti-hFVIII titer of 54 Bethesda Units (BU)/mL. Twenty-six patients (52%) had a detectable anti-rpFVIII with a median titer of 2 BU/mL (IQR, 0.86-5.8; range, 0.55-34; Table 1).

### 3.3 Response to rpFVIII

In our cohort, 42 patients received rpFVIII. The median first dose received was 99 U/kg (IQR, 93-104; range, 5.9-155). Twenty-two patients with AHA with baseline cross-reacting inhibitors received rpFVIII to treat bleeding. Median recovery of FVIII activity for all 42 patients was 1.2 IU/mL per U/kg infused (IQR, 0.54-1.7; range, 0-2.5; Table 1).

In the group of patients without a detectable cross-reacting antibody, the median rise in FVIII activity was 1.3 IU/mL per IU/kg infused (IQR, 0.6-2). In all patients with a detectable anti-rpFVIII titer, the median rise in FVIII level was 0.54 IU/mL per IU/kg (IQR, 0-1.8). In those with an inhibitor titer less than 5 BU/mL, the median response was increased to 0.82 IU/mL per IU/kg, and for those with titers ≥ 5 BU/mL, the median response was 0.12 IU/mL per IU/kg (Figure).

**Figure:**
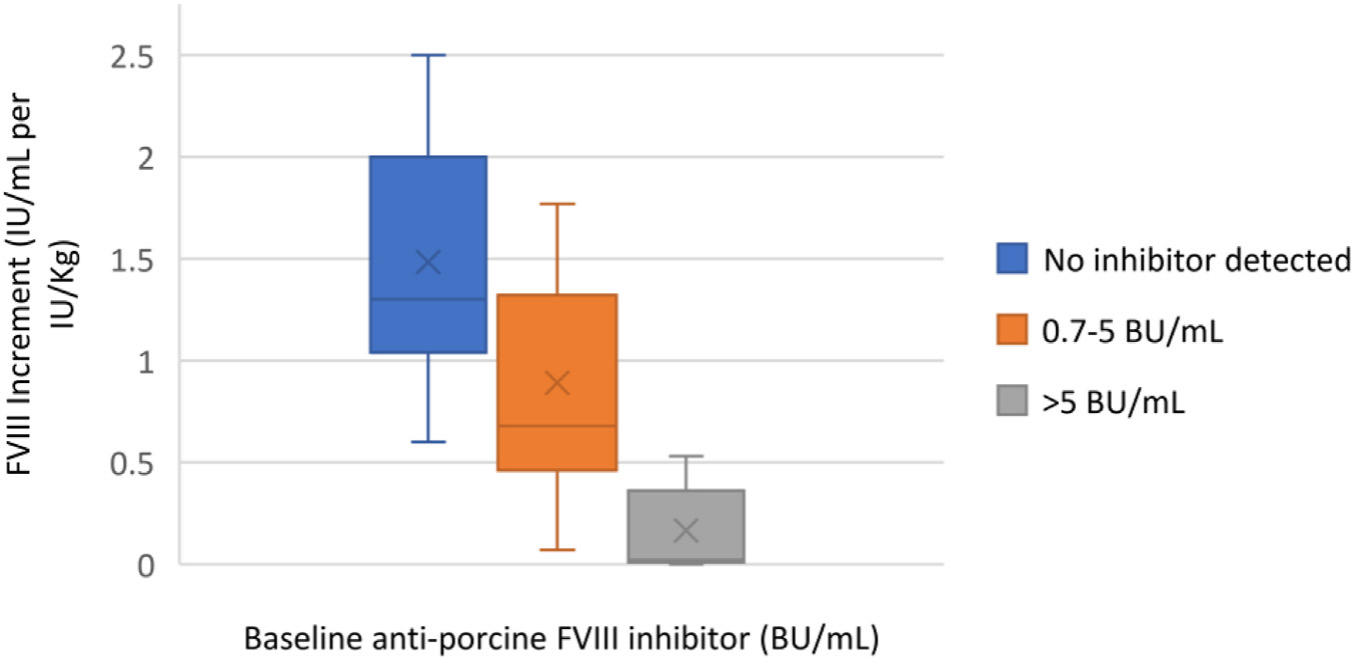
Factor (F)VIII response immediately after the first dose of Obizur (Takeda) in relation to antiporcine FVIII inhibitor level. The x-axis represents the FVIII increment (IU/mL per IU/kg) after the first dose of recombinant B-domain-deleted porcine FVIII (rpFVIII; Obizur) was administered. The colored bars demonstrate varying FVIII increments based on the baseline antiporcine FVIII inhibitor. The blue bar demonstrates FVIII increment after the first dose of rpFVIII was administered when no baseline inhibitor was present. The orange bar represents the FVIII increment in response to rpFVIII with a baseline inhibitor titer between 0.7 and 5 Bethesda Units (BU)/mL. The gray bar represents the FVIII increment in response to rpFVIII with a baseline inhibitor titer > 5 BU/mL.

### 3.4 Treatment duration

The duration of treatment with rpFVIII ranged between 1 and 21 days (median, 7 days). The average dose of rpFVIII in Units/kg/d was 135, ranging from 37 to 311. Patients with a higher baseline anti-rpFVIII antibody received a higher dose. Those with an inhibitor titer ≤ 5 BU/mL received an average dose of 163 Units/kg/d, ranging from 64 to 235. The average dose for those with an inhibitor titer >5 BU/mL was 218 Units/kg/d, ranging from 99 to 310.

### 3.5 Follow-up

Twenty-nine of the patients who received rpFVIII had repeat testing for inhibitors during their hospital stay. Once discharged, patients who were seen in clinic for follow-up were subsequently tested for anti-hFVIII and anti-rpFVIII. For these patients, the baseline anti-hFVIII median level was 84 BU/mL, which decreased to a median of 61 BU/mL on the first repeat after a median of 8 days (range, 2-22). Four AHA patients without a baseline antiporcine FVIII inhibitor who received rpFVIII treatment developed a *de novo* inhibitor with a median titer of 1.5 BU/mL (range, 0.75-12) after a median of 21 days (range, 9-134). Three AHA patients had a rise in baseline anti-rpFVIII titer after exposure to rpFVIII after a median of 15 days (range, 10-15; Table 2).

**Table 2:** Patients who developed an increase in their antiporcine factor VIII inhibitor titer after exposure to recombinant B-domain-deleted porcine factor VIII.

Patient ID	1	2	3
Baseline antiporcine FVIII titer (pBU/mL)	13	4	2
Baseline anti-hFVIII titer (BU/mL)	384	563	309
Time to first increase of antiporcine FVIII (d)	15	15	10
Time to first increase of anti-hFVIII (d)	No increase	36	10
Titer of anti-hFVIII post exposure (BU/mL)	346	666	393
Titer of antiporcine FVIII post exposure (pBU/mL)	29	7	4
Percentage of increase in antiporcine FVIII titer (%)	123	75	100

BU, Bethesda Units; FVIII, factor VIII; hFVIII, human factor VIII.

## 4 Discussion

This study describes what we believe to be the largest North American cohort of patients treated with rpFVIII. RpFVIII offers a therapeutic option for bleeding in patients with AHA due to immunologic differences in the A2 and C2 domains of rpFVIII while maintaining sufficient hFVIII homology to provide effective hemostasis. An additional benefit is the possibility of monitoring the FVIII response with conventional activity assays.

In our cohort, 52% of patients with AHA had detectable baseline cross-reacting inhibitors to rpFVIII. This is a larger proportion of patients compared with previously published data (35% in the phase 2/3 trial) [1,5].

In our cohort, 33 patients were treated with a first dose of rpFVIII of approximately 100 U/kg. The immediate FVIII recovery varied widely depending on the presence and titer of baseline cross-reacting inhibitors. In the phase 2/3 trial, the initial dose was 200 U/kg [5]. Our study suggests that an initial rpFVIII dose of 100 U/kg appears sufficient to overcome baseline inhibitors up to a titer of 5 pBU/mL, in agreement with the case series reported by Tarantino et al. [9]. In addition, the immediate posttreatment FVIII level was predictive of the presence and strength of the anti-rpFVIII inhibitor. Lesser incremental increases in FVIII activity were seen in those patients with pBU titers greater than 5 BU/mL. We were able to achieve therapeutic FVIII activity levels with adjusted dosing regimens even in the presence of high inhibitor titers (data not shown) [9].

Twenty-nine (69%) patients receiving rpFVIII had repeat testing for inhibitors during their hospital stay or after discharge. This ranged between weeks to months after the initial documentation of inhibitors. Of the 12 patients without a baseline inhibitor, 4 patients with AHA (33%) who received rpFVIII treatment developed a *de novo* anti-rpFVIII inhibitor with a median titer of 1.5 BU/mL (range, 0.75-12) after a median of 21 days (range, 9-134). This is in comparison with the phase 2/3 trial, in which 5 out of 28 patients (18%) were found to have a newly detectable anti-rpFVIII antibody. This discrepancy may be explained by a longer follow-up period in our study compared with the phase 2/3 trial, in which the follow-up period was 90 days. In our study, 1 patient developed an anti-rpFVIII inhibitor on day 134. In addition, of the 18 patients with a baseline inhibitor in our study, the titers increased in only 3 patients with AHA (17%) after exposure to rpFVIII after a median of 15 days (range, 10-15).

## 5 Conclusion

In conclusion, we found that 52% of patients with AHA had a detectable baseline cross-reacting inhibitor, which is a higher frequency than previously described. An initial dose of 100 U/kg of rpFVIII appeared sufficient to achieve therapeutic FVIII levels in patients without a detectable baseline cross-reacting anti-rpFVIII inhibitor. The presence of anti-rpFVIII inhibitors at baseline was associated with decreased FVIII activity levels obtained in response to rpFVIII infusion. Finally, in our cohort, exposure to rpFVIII induced the development of *de novo* anti-rpFVIII inhibitors more frequently than previously reported.

## References

[1] Türkantoz H., Königs C., Knöbl P., Klamroth R., Holstein K., Huth-Kühne A. (2020). Cross-reacting inhibitors against recombinant porcine factor VIII in acquired hemophilia A: data from the GTH - AH 01 / 2010 study. J Thromb Haemost.

[2] Freedman J., Mody M., Lazarus A.H., Dewar L., Song S., Blanchette V.S. (2002). Platelet activation and hypercoagulability following treatment with porcine factor VIII (HYATE:C). Am J Hematol.

[3] Soucie J.M., Erdman D.D., Evatt B.L., Anderson L.J., Török T.J., El-Jamil M. (2000). Investigation of porcine parvovirus among persons with hemophilia receiving Hyate:C porcine factor VIII concentrate. Transfusion.

[4] Dargaud Y., Escuriola-Ettingshausen C. (2021). Recombinant porcine factor VIII: Lessons from the past and place in the management of hemophilia A with inhibitors in 2021. Res Pract Thromb Haemost.

[5] Kruse-Jarres R., St-Louis J., Greist A., Shapiro A., Smith H., Chowdary P. (2015). Efficacy and safety of OBI-1, an antihaemophilic factor VIII (recombinant), porcine sequence, in subjects with acquired haemophilia A. Haemophilia.

[6] Elbaz C., Pavenski K., Chaudhry H., Teitel J.M., Sholzberg M. (2019). The frequency and effect of baseline cross-reacting and *de novo* inhibitors to recombinant porcine FVIII in patients with congenital and acquired hemophilia A. Blood.

[7] Verbruggen B., Novakova I., Wessels H., Boezeman J., van den Berg M., Mauser-Bunschoten E. (1995). The Nijmegen modification of the Bethesda assay for factor VIII:C inhibitors: improved specificity and reliability. Thromb Haemost.

[8] (2021). St. Michael’s Department of Laboratory Medicine Division of Hematopathology. Nijmegen-Bethesda FVIII Inhibitor Assay. https://stmichaelshospital.com/mirror-hosts/stmichaelshospital.com/programs/labs/tests/F/nijmegen-bethesda-fviii.php.

[9] Tarantino M.D., Cuker A., Hardesty B., Roberts J.C., Sholzberg M. (2017). Recombinant porcine sequence factor VIII (rpFVIII) for acquired haemophilia A: practical clinical experience of its use in seven patients. Haemophilia.

